# Effects of Ozone Fumigation on the Main Postharvest Pathogenic Fungi *Penicillium* sp. and the Storage Quality of Blueberry in Majiang County, China

**DOI:** 10.3389/fpls.2022.898994

**Published:** 2022-05-27

**Authors:** Wenneng Wu, Sen Cao, Haijiang Chen, Lixiu Ruan, Qiqing Lei, Su Xu, Jiangkuo Li

**Affiliations:** ^1^Food and Pharmaceutical Engineering Institute, Guiyang University, Guiyang, China; ^2^National Engineering Technology Research Center for Preservation of Agricultural Products/Key Laboratory of Storage of Agricultural Products, Ministry of Agriculture and Rural Affairs/Tianjin Key Laboratory of Postharvest Physiology and Storage of Agricultural Products, Tianjin, China

**Keywords:** ozone fumigation, inhibitory effect, *Penicillium* sp., blueberry, storage quality

## Abstract

In this study, the fungus *Penicillium* sp. was isolated from rotting postharvest blueberry fruits at different storage stages and identified into genera. Inoculation of this strain on the surface of fresh fruits was able to cause rotting. The strain was then used as a reference strain to test the chemical control effect of ozone fumigation during storage. The results showed that ozone fumigation had an obvious inhibitory effect on *Penicillium* sp. in a dose- and time-dependent manner. Meanwhile, ozone fumigation treatment could prevent the loss of fruit firmness, slow down the decrease of soluble solids, total phenolics, and anthocyanins, and maintain a lower activity of PPO and higher activities of POD and CAT. As far as we know, this is the first report on the effects of ozone fumigation on the postharvest pathogenic fungi *Penicillium* sp. and on the storage quality of postharvest blueberry collected from Majiang County, Guizhou province, China.

## Introduction

Blueberry (*Vaccinium*, section *Cyanococcus*), one of the most valuable fruits, originated in North America and is widely distributed around the world because of its potential health benefits (Rimando et al., 2004; Giacalone et al., 2011). However, blueberry is quite susceptible to several diseases (Li et al., 2010). Once infected by the storage pathogens, the pathogens can soften the walls of the blueberry fruits and transmit to the neighboring blueberry fruits, resulting in a significant economic loss (Yang et al., 2014). It is of great social and economic significance to prolong blueberry storage life and the marketing period. To date, many measures have been taken to control the blueberry postharvest diseases (Hanson et al., 1993; Almenar et al., 2008; Perkins-Veazie et al., 2008; Angeletti et al., 2010; Wang and Chen, 2010; Yang et al., 2014; Vieira et al., 2016; Chu et al., 2018). On the other hand, consumers' demand for high-quality products makes it necessary to limit the use of chemical fungicides, which makes the postharvest fungal disease control more challenging (El Ghaouth et al., 1992). Therefore, it is valuable to use natural alternatives to prevent blueberry postharvest diseases.

Ozone, a well-known strong oxidizing agent, generally safe to use, has been used by the food industry as an antimicrobial agent for many years due to its rapid decomposition to harmless oxygen, leaving no residues in food and the environment (Muthukumarappan et al., 2000; Pryor, 2001; Tzortzakis et al., 2007). In 2002, the Food and Drug Administration (FDA) approved the use of ozone to prevent postharvest diseases of fruits and vegetables. In the past few decades, ozone has been tested in different operative conditions as a postharvest treatment against postharvest diseases (Bialka and Demirci, 2007; Alexandre et al., 2011; Pangloli and Hung, 2013; Contigiani et al., 2018). However, there are no reports on the effects of ozone fumigation treatment on *Penicillium* sp. isolated from postharvest blueberry.

The aim of this work was to evaluate the effects of ozone fumigation treatment at different doses and durations on *Penicillium* sp. which was isolated from the rotting postharvest blueberry collected from Majiang County, Guizhou province, China. Experimental results indicated that ozone fumigation had an obvious inhibitory effect on *Penicillium* sp., and could significantly inhibit the conidial germination, spore production, mycelium growth, and colony expansion and thus was able to reduce the pathogenicity of *Penicillium* sp. In addition, this work also found that ozone fumigation treatment could delay the spoilage, prevent the loss of fruit firmness, inhibit the decrease of soluble solids, total phenolics, and anthocyanins, and maintain lower polyphenol oxidase (PPO) activity and higher peroxidase (POD) and catalase (CAT) activities of blueberry to prolong the blueberry storage life.

## Materials and Methods

### Materials

Blueberry fruits were hand-harvested from a 10- to 11-year-old blueberry plant in the Majiang County, Guizhou Province, China, in July 2020. The postharvest blueberry fruits were immediately transported to the lab of Guiyang University by car at 20°C. The blueberry fruits of uniform size and color and without physical damage were selected for subsequent experiments.

### Fungi Isolation, Purification, and Pathogenicity Test

Decayed blueberry fruits were surface-sterilized using sterile 75% ethanol, washed with sterile distilled water three times, excised the infected tissues with a sterile scalpel, plated the infected tissues on the sterile potato-dextrose agar (PDA) plates, and then incubated the PDA plates in an incubator at 28°C for 3 days. All isolations isolated from the rotting postharvest blueberry fruits were sub-cultured on new PDA plates using the single spore technique (Kwon et al., 2011). Individual colonies were isolated and sub-cultured twice to ensure purity, and then the spores were harvested in sterile distilled water for later use. Pathogenicity tests were performed by spraying a 1.0 × 10^6^ conidia/L conidial suspension with 0.1% Tween 20 (v/v) on the surface of the blueberry fruits, and the blueberry fruits were incubated at 28°C with 95% relative humidity (RH) for 7 days (Kwon et al., 2011). Blueberry fruits sprayed with sterile distilled water were used as control.

### Morphological and Molecular Identification

Identification of the individual colonies at the genus level was carried out by morphological and molecular methods (Qiu et al., 2014). Briefly, individual colonies were inoculated on the PDA medium at 28°C for 7 days and identified by both the eye and a Model EX30 inverted microscopy (Ningbo Shunyu Tech. Co. LTD, Zhejiang, China).

Fungal mycelia (~100 mg) were collected for DNA extraction using the TIANamp fungal DNA kit (Tiangen-Biotech Co. LTD, Beijing, China) (Kwon et al., 2011). Polymerase chain reactions (PCR) were conducted using a Premix Taq Ver. 2.0 plus dye kit (Takara, Dalian, China) according to the manufacturer's instructions with the universal primer ITS1 (5′-TCCGTAGGTGAACCTGCGG-3′) and ITS4 (5′-TCCTCCGCTTATTGATATGC-3′) and the following amplification program: 95°? for 5 min; 30 cycles of 94°? for 30 s, 55°? for 30 s, and 72°C for 1 min; 72°? for 10 min. After that, the PCR products were sequenced at Sangon Corporation (Shanghai, China) and searched for sequence similarity using the National Center for Biotechnology Information (NCBI) blast program (http://www.ncbi.nlm.nih.gov/blast.cgi). Phylogenetic trees were constructed by the neighbor-joining method using the MEGA 6.0 software.

### Preparation of Fungal Spore Suspensions

Spores were obtained by cultivating the tested fungi on the PDA plates for 7 days and then harvested by adding sterile distilled water to the Petri plates and removing mycelia by filtering through four layers of sterile cheesecloth. The spore suspensions were adjusted to ~1.0 × 10^6^ conidia/L concentration with a hemocytometer for later use (Jia et al., 2016).

### Ozone Fumigation System

Different concentrations of ozone were introduced into a plastic chamber (112.0 cm length × 47.5 cm width × 42.5 cm height) by a Model SH-802-4G/H ozone generator (Guangzhou Shenghuan Environmental Protection Tech. Co. LTD, Guangzhou, China).

### *In vitro* Antifungal Effect

#### Effect on the Number of Colonies

The 1.0 × 10^6^ conidia/L concentration spore suspension (2.5 μl) was pipetted onto the PDA plates and then distributed evenly with a spreader. After that, the PDA plates were placed in the ozone fumigation system and exposed to the ozone with different concentrations of 0, 50, 100, 150, and 200 mg/m^3^ for 15, 30, 45, and 60 min, respectively. Then, the PDA plates were incubated in the sterile chamber at 28°C for 2 days. The inhibition rates of different ozone concentrations were calculated using the following equation, where G_t_ is the number of individual colonies in the treatment group and G_c_ is the number of individual colonies in the control group.


$$\begin{array}{l} \text{I1.4em;text-indent:-1.4n1.4em;text-indent:-1.4h1.4em;text-indent:-1.4i1.4em;text-indent:-1.4b1.4em;text-indent:-1.4i1.4em;text-indent:-1.4t1.4em;text-indent:-1.4i1.4em;text-indent:-1.4o1.4em;text-indent:-1.4n1.4em;text-indent:-1.4 1.4em;text-indent:-1.4r1.4em;text-indent:-1.4a1.4em;text-indent:-1.4t1.4em;text-indent:-1.4e1.4em;text-indent:-1.4 1.4em;text-indent:-1.4I1.4em;text-indent:-1.4 }\left(\text{\%}\right)=\left({\text{G}}_{\text{c}}-{\text{G}}_{\text{t}}\right)/{\text{G}}_{\text{c}}\times \;100\text{\%} \end{array}$$

#### Effect on the Colony Diameter

The 1.0 × 10^6^ conidia/L concentration spore suspension (2.5 μL) was transferred to the center of PDA plates. After exposing to ozone with different concentrations of 0, 50, 100, 150, and 200 mg/m^3^ for 15, 30, 45, and 60 min, respectively, the PDA plates were then incubated in the sterile chamber at 28°C. After 3 days of incubation, the colony diameters were determined by the cross-sectional method using a vernier caliper. The inhibition rates were calculated using the following equation, where L_t_ is the colony diameter length in the treatment group and L_c_ is the colony diameter length in the control group.


$$\begin{array}{l} \text{I1.4em;text-indent:-1.4n1.4em;text-indent:-1.4h1.4em;text-indent:-1.4i1.4em;text-indent:-1.4b1.4em;text-indent:-1.4i1.4em;text-indent:-1.4t1.4em;text-indent:-1.4i1.4em;text-indent:-1.4o1.4em;text-indent:-1.4n1.4em;text-indent:-1.4 1.4em;text-indent:-1.4r1.4em;text-indent:-1.4a1.4em;text-indent:-1.4t1.4em;text-indent:-1.4e1.4em;text-indent:-1.4 1.4em;text-indent:-1.4I1.4em;text-indent:-1.4 }\left(\text{\%}\right)=\left({\text{L}}_{\text{c}}-{\text{L}}_{\text{t}}\right)/{\text{L}}_{\text{c}}\times \;100\text{\%} \end{array}$$

#### Effect on the Mycelial Growth

The effects on mycelial growth were assessed according to a described method (Jia et al., 2016). The 1.0 × 10^6^ conidia/L concentration spore suspension (50 ml) was placed in a 150 ml flask and exposed to ozone with different concentrations of 0, 50, 100, 150, and 200 mg/m^3^ for 15, 30, 45, and 60 min, respectively. Then, 2 ml of each spore suspension was transferred into the 150 ml flask containing 100 ml potato-dextrose (PD) medium and incubated in the chamber at 28°C. After 6 days of inoculation, the effects on mycelial growth were determined by measuring the dry weight of colony mass using electronic scales. The percentage inhibitions of mycelial growth were calculated using the following equation, where m_t_ is the mycelial mass of the treatment group and m_c_ is the mycelial mass of the control group.


$$\begin{array}{l} \text{I1.4em;text-indent:-1.4n1.4em;text-indent:-1.4h1.4em;text-indent:-1.4i1.4em;text-indent:-1.4b1.4em;text-indent:-1.4i1.4em;text-indent:-1.4t1.4em;text-indent:-1.4i1.4em;text-indent:-1.4o1.4em;text-indent:-1.4n1.4em;text-indent:-1.4 1.4em;text-indent:-1.4r1.4em;text-indent:-1.4a1.4em;text-indent:-1.4t1.4em;text-indent:-1.4e1.4em;text-indent:-1.4 }\left(\text{\%}\right)=\left({\text{m}}_{\text{c}}-{\text{m}}_{\text{t}}\right)/{\text{m}}_{\text{c}}\times \;100\text{\%} \end{array}$$

#### Effect on the Spore Production

A total of 2.5 μl spore suspension (1.0 × 10^6^ conidia/L) were distributed evenly onto the PDA mediums with a spreader and then exposed to different ozone concentrations of 0, 50, 100, 150, and 200 mg/m^3^ for 15, 30, 45, and 60 min, respectively. After 7 days of incubation in the chamber at 28°C, the spore number was counted using a hemocytometer. The spore germination inhibition rates were calculated using the following equation, where n_t_ is the number of spore production number of the treatment group and n_c_ is that of the control group.


$$\begin{array}{l} \text{I1.4em;text-indent:-1.4n1.4em;text-indent:-1.4h1.4em;text-indent:-1.4i1.4em;text-indent:-1.4b1.4em;text-indent:-1.4i1.4em;text-indent:-1.4t1.4em;text-indent:-1.4i1.4em;text-indent:-1.4o1.4em;text-indent:-1.4n1.4em;text-indent:-1.4 1.4em;text-indent:-1.4r1.4em;text-indent:-1.4a1.4em;text-indent:-1.4t1.4em;text-indent:-1.4e1.4em;text-indent:-1.4 }\left(\text{\%}\right)=\left({\text{n}}_{\text{c}}-{\text{n}}_{\text{t}}\right)/{\text{n}}_{\text{c}}\times \;100\text{\%} \end{array}$$

#### Effect on the Spore Germination

The effects of ozone fumigation treatment at different doses and exposure times on spore germination were assayed using Ong's method (Ong and Ali, 2015). Aliquots of 100 μl of spore suspension (1.0 × 10^6^ conidia/L) were transferred to the PDA plates and then exposed to different ozone concentrations of 0, 50, 100, 150, and 200 mg/m^3^ for 15, 30, 45, and 60 min, respectively. After 12 h incubation in the chamber at 28°C, the spore germination rate was calculated. The spore germination rate was calculated using the following equation, where G_t_ is the germination rate of the treatment group while G_c_ is that of the control group.


$$\begin{array}{l} \text{I1.4em;text-indent:-1.4n1.4em;text-indent:-1.4h1.4em;text-indent:-1.4i1.4em;text-indent:-1.4b1.4em;text-indent:-1.4i1.4em;text-indent:-1.4t1.4em;text-indent:-1.4i1.4em;text-indent:-1.4o1.4em;text-indent:-1.4n1.4em;text-indent:-1.4 1.4em;text-indent:-1.4r1.4em;text-indent:-1.4a1.4em;text-indent:-1.4t1.4em;text-indent:-1.4e1.4em;text-indent:-1.4 }\left(\text{\%}\right)=\left({\text{G}}_{\text{c}}-{\text{G}}_{\text{t}}\right)/{\text{G}}_{\text{c}}\times \;100\text{\%} \end{array}$$

### Effects on Storage Quality of Postharvest Blueberry

#### Ozone Fumigation Treatment

To investigate the effects of ozone fumigation on the postharvest blueberry fruits storage quality, freshly harvested fruits, pre-exposed to ozone 200 mg/m^3^ concentration for 60 min in the ozone fumigation system, were used for the subsequent storage experiments.

#### Decay Rate

After ozone fumigation treatment, the blueberry fruits were stored for a total of 80 days at 4°C and sampled at a 20-day interval. The fruits with visible mold were considered decayed. The decay rate analysis was performed in triplicates with 20 fruits randomly sampled for each replicate.

#### Fruit Firmness

The blueberry fruit firmness was measured by a TA.XT Plus Texture Analyzer (Stable Micro Systems, Godalming, United Kingdom) according to Angeletti's method (Angeletti et al., 2010). For each treatment, measurement was performed on 10 fruits.

#### Soluble Solids, Total Phenolics, and Anthocyanins Contents Assays

To assess the influence of ozone fumigation on internal fruit quality, the soluble solids contents of the sampled blueberry fruits at different time intervals were determined using a PAL-1 digital refractometer (ATAGO, Tokyo, Japan) according to Zhang's method (Zhang et al., 2019). Total phenolics contents were determined with a Folin–Ciocalteu reagent according to Buckow's report (Buckow et al., 2010). Anthocyanin contents were determined using the UV-Vis spectrophotometer (UV-2550, SHIMADZU, Japan) method (Buckow et al., 2010; You et al., 2011). Each assay was performed 10 times.

#### Determination of PPO, POD, and CAT Activities

After ozone fumigation, the PPO, POD, and CAT activities of the sampled blueberry fruits were measured using the corresponding enzyme assay reagent kits (Suzhou Comin Bioengineering Institute, Suzhou, China).

### Statistical Analysis

All data represented in this study are analyzed using SPSS version 23 software (IBM, NY, USA). The *p*-value lower than 0.05, analyzed by statistical significance, is considered a significant difference.

## Results and Discussion

### Fungal Isolation and Identification

**Figure 1:**
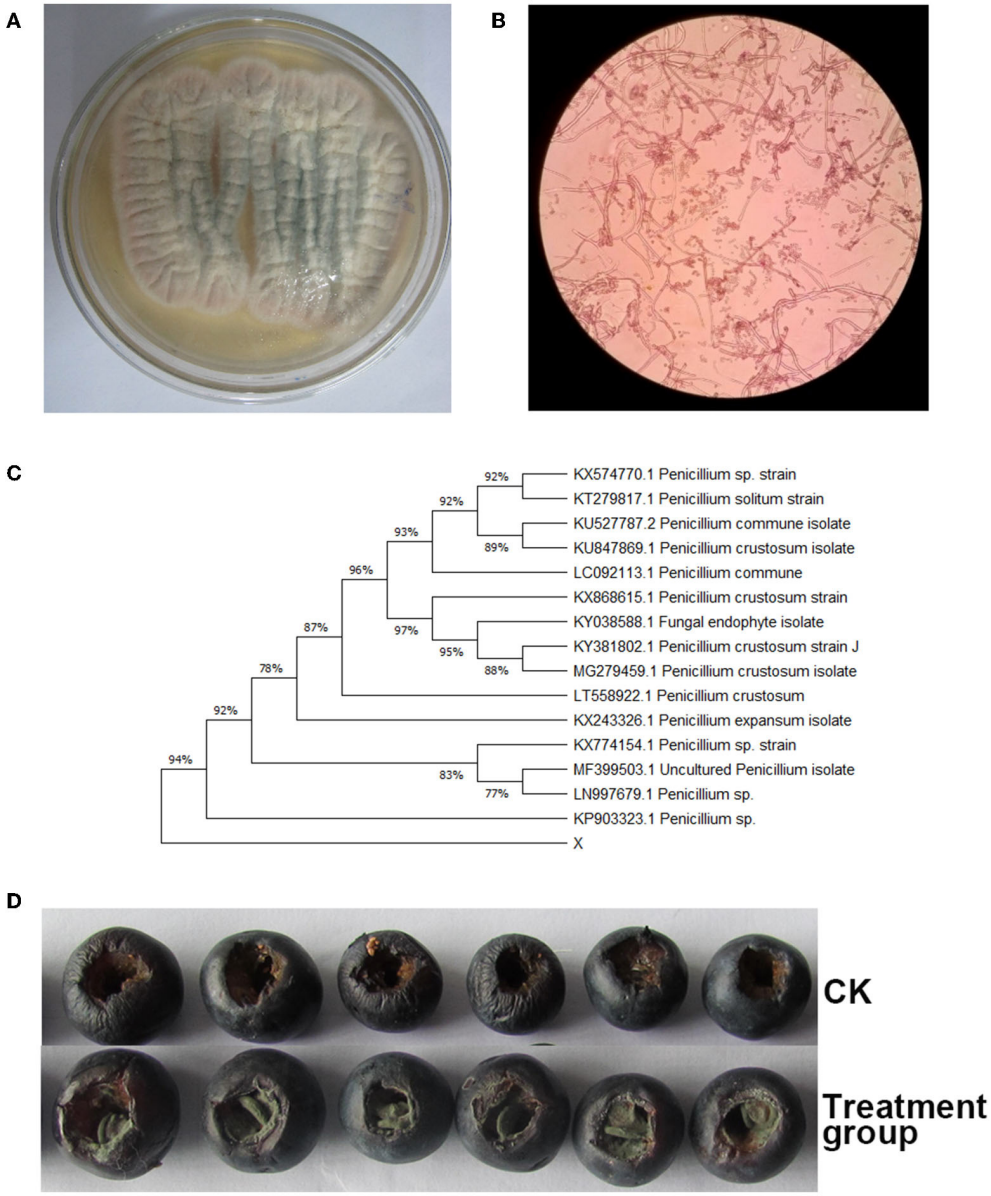
Morphology of Y7 isolate cultures on PDA. **(A)** observe the surface of the colony, **(B)** structural characteristics of conidiophores, **(C)** UPGMA tree based on sequence analysis of internal transcribed spacer sequences, and **(D)** serious symptoms in pathogenicity tests.

Figures 1A,B showed that the fungal strain Y7 appearance was white in the adjustment period, pink in the exponential period and stable period, and black in the decaying period. Meanwhile, based on the morphological characteristics and ITS sequences (Figure 1C), the fungal strain Y7 isolated from rotting postharvest blueberry fruits was classified as *Penicillium* sp. (Accession No. KP903323.1) with a similarity of 94%. In addition, 7 days after inoculation, Figure 1D showed that serious symptoms of blue mold were observed on the blueberry fruits surface. Then, the appeared fungus was isolated and showed identical morphological characteristics with the original culture, demonstrating that the pathogen that caused rotting on the surface of postharvest blueberry fruits is *Penicillium* sp. The results here imply that the fungus *Penicillium* sp. Y7 can cause the rotting of blueberry fruits. Unlike frequently identified fungal pathogens like *Botrytis cinerea, Alternaria* spp., *Colletotrichum* spp., reporting of *Penicillium* as causing agent of blueberry fruits decay is relatively rare although Penicillium has been found on diseased blueberry fruits (Tournas and Katsoudas, 2005; Mehra et al., 2013; Ramos-Bell et al., 2021).

### *In vitro* Antifungal Effect Assays

**Figure 2:**
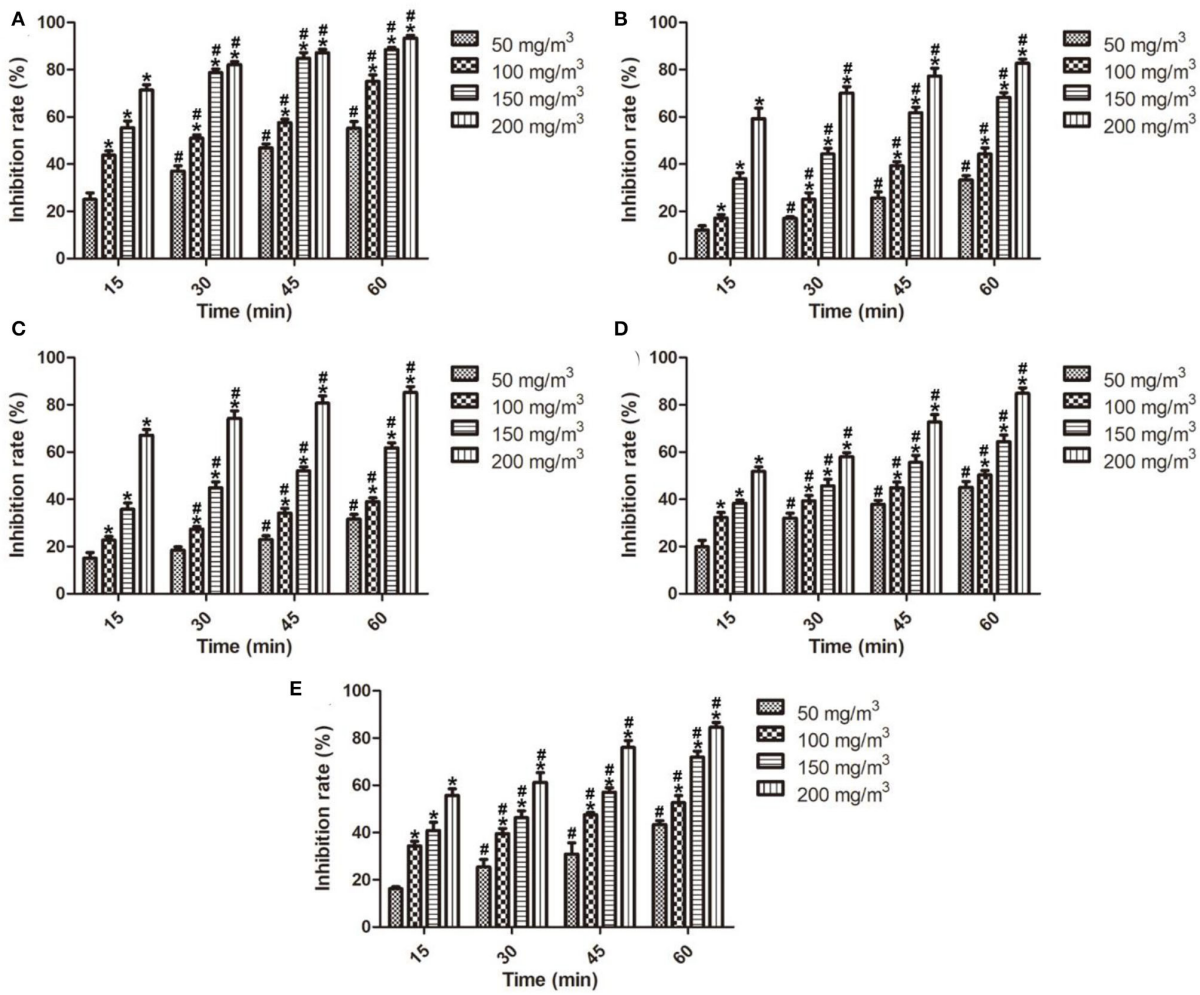
*In vitro* antifungal effects of ozone fumigation treatment on *Penicillium* sp. at different ozone concentrations and treatment time. **(A)** *in vitro* antifungal effect on the number of colonies, **(B)** *in vitro* antifungal effect on the colony diameter, **(C)** *in vitro* antifungal effect on the mycelial growth, **(D)** *in vitro* antifungal effect on the spore germination, and **(E)** *in vitro* antifungal effect on the spore germination. “*” indicated the inhibition rates of ozone fumigation treatment with a significant difference at *p* < 0.05 compared with the inhibition rates at 50 mg/m^3^. “#” indicated the inhibition rates of ozone fumigation treatment with a significant difference at *p* < 0.05 compared with the inhibition rates at 15 min.

Fruits spoilage is a complex process and excessive amounts of fruits are lost due to fungal contamination (Gram et al., 2002). *Penicillium* sp. is a common pathogen in pome, stone, citrus, and grapefruits during storage because the fungus can even grow at freezing temperature (Dukare et al., 2020). Literature reported that ozone can disrupt fungal cells by oxidizing sulfhydryl and amino acid groups of enzymes or by attacking the polyunsaturated fatty acids on the cell wall (Afsah-Hejri et al., 2020). In the past few years, ozone had been evaluated for postharvest diseases control and other storage uses. In this study, the *in vitro* antifungal effects of ozone fumigation treatment on the number of colonies, colony diameter, mycelial growth, spore germination, and spore production of *Penicillium* sp. At different ozone concentrations and treatment time were determined and are illustrated in Figure 2. It showed that the number of colonies, colony diameter, mycelial growth, spore germination, and spore germination of *Penicillium* sp. were significantly reduced by ozone fumigation treatment with dose- and time-dependence, and the inhibition rates reached 93.40% (Figure 2A), 82.70% (Figure 2B), 85.17% (Figure 2C), 84.91% (Figure 2D), and 84.58% (Figure 2E).

### Physicochemical Properties

#### Decay Rate and Fruits Firmness Assays

**Figure 3:**
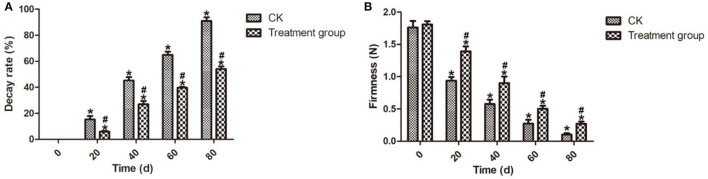
Effects on the decay rates and fruits firmness of the postharvest blueberries after ozone fumigation treatment. **(A)** Decay rate and **(B)** Fruits firmness. “*” indicated the decay rates and fruits firmness of ozone fumigation treatment with a significant difference at *p* < 0.05 compared with the decay rates and fruits firmness at 0 d. “#” indicated the inhibition rates of ozone fumigation treatment with a significant difference at *p* < 0.05 compared with the CK group.

Firmness is the most important quality attribute that affects the consumer attraction and marketing of postharvest blueberry fruits (Sun et al., 2014). In this study, compared with the control group (CK), the decay rate (Figure 3A) after ozone fumigation treatment for 80 days at 4°C was 54.03%, which was lower than the CK sample of 36.91%. Meanwhile, Figure 3B shows that, comparing to the CK samples during the storage, ozone fumigation treatment can delay the descendant tendency of the firmness of the postharvest blueberry fruits. After storage for 80 days at 4°C, the postharvest blueberry fruits treated with ozone fumigation can maintain better firmness with a value of 0.27 N, while that of the CK sample was only 0.10 N. Similar effect of ozone on the firmness of other fresh commodities such as bell pepper has been observed (Alwi and Ali, 2016).

#### Soluble Solids, Total Phenolics, and Anthocyanins Contents Assays

**Figure 4:**
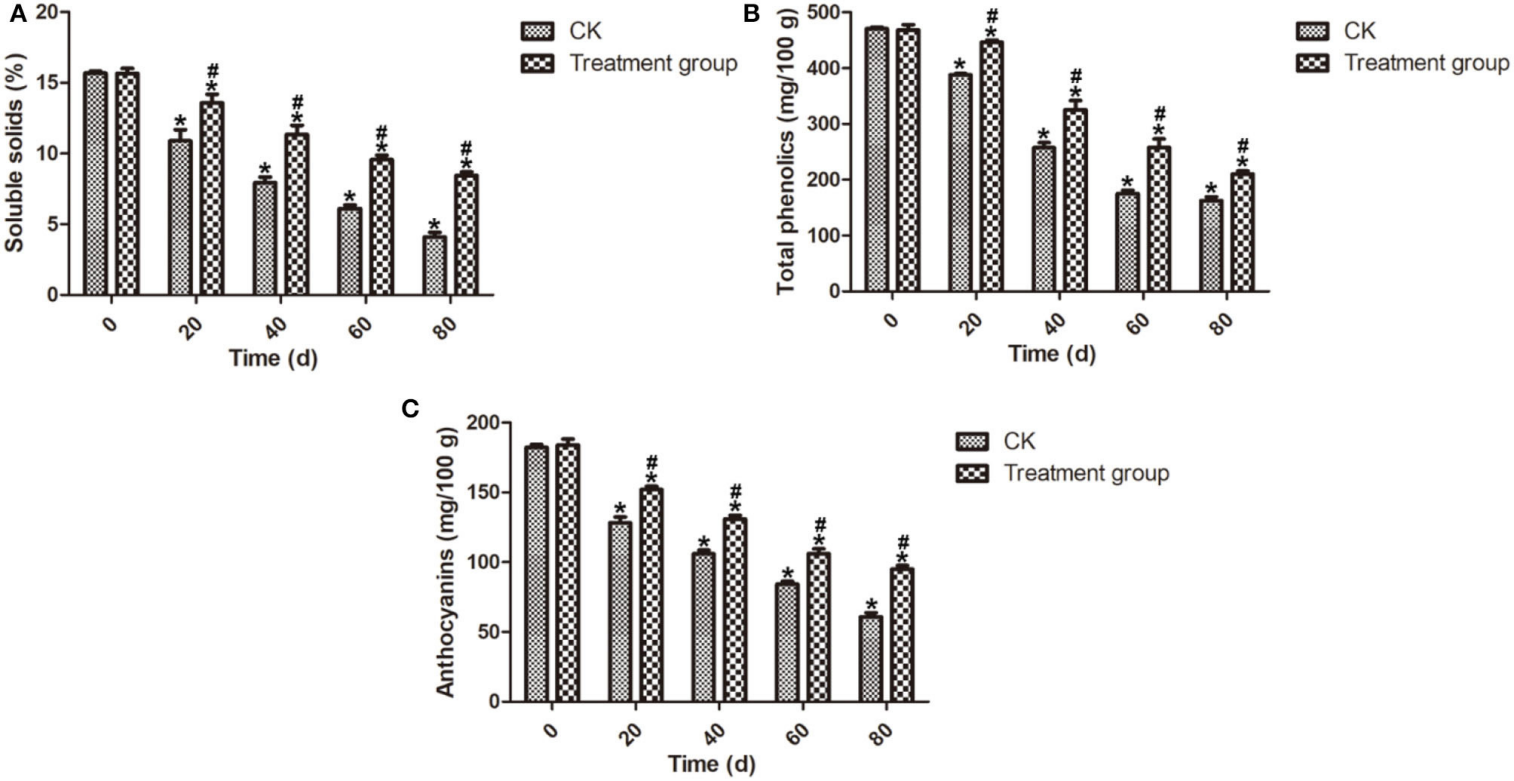
Effects on the soluble solids, total phenolics, and anthocyanins contents of the postharvest blueberries after ozone fumigation treatment. **(A)** Effect on the soluble solids content, **(B)** effect on the total phenolics content, and **(C)** effect on the anthocyanins content. “*” indicated the decay rates and fruits firmness of ozone fumigation treatment with a significant difference at *p* < 0.05 compared with the decay rates and fruits' firmness at 0 d. “#” indicated the inhibition rates of ozone fumigation treatment with a significant difference at *p* < 0.05 compared with the CK group.

Soluble solids content is an important index of intrinsic quality and fruit ripeness (Han et al., 2017). Phenolic compounds present in the blueberry fruits' skin have antioxidant properties and are largely beneficial to the health of the blueberry fruits, depending on the physiological state and the storage conditions (Gao and Mazza, 1994; Michalska and Łysiak, 2015). Anthocyanins have been shown to improve the antioxidant capacity of berry fruits (Beattie et al., 2005). A significant decrease in the soluble solids, total phenolic, and anthocyanin contents of all samples was observed and the results are listed in Figure 4. As shown in Figure 4A, ozone fumigation treatment could delay the decrease of the soluble solids content compared to the CK samples during the storage. At the end of storage (80 d), the soluble solids content of the postharvest blueberry fruits after ozone fumigation treatment at 4°C is 8.44%, while that of the CK samples was only 4.10%. Meanwhile, Figures 4B,C showed that, at the end of the storage (80 d), the content values of the total phenolics and anthocyanins of the postharvest blueberry fruits after ozone fumigation treatment were 210.17 and 95.17 mg/100 g, while those of the CK samples were 162.62 and 60.83 mg/100 g. The results here showed that ozone only slows down the rate of decrease of total phenolics in blueberry fruits, while in the case of the mushroom *Ganoderma lucidum*, ozone treatment can increase the total phenolics and flavonoids (Sudheer et al., 2016).

#### Enzyme Activity of PPO, POD, and CAT Assays

**Figure 5:**
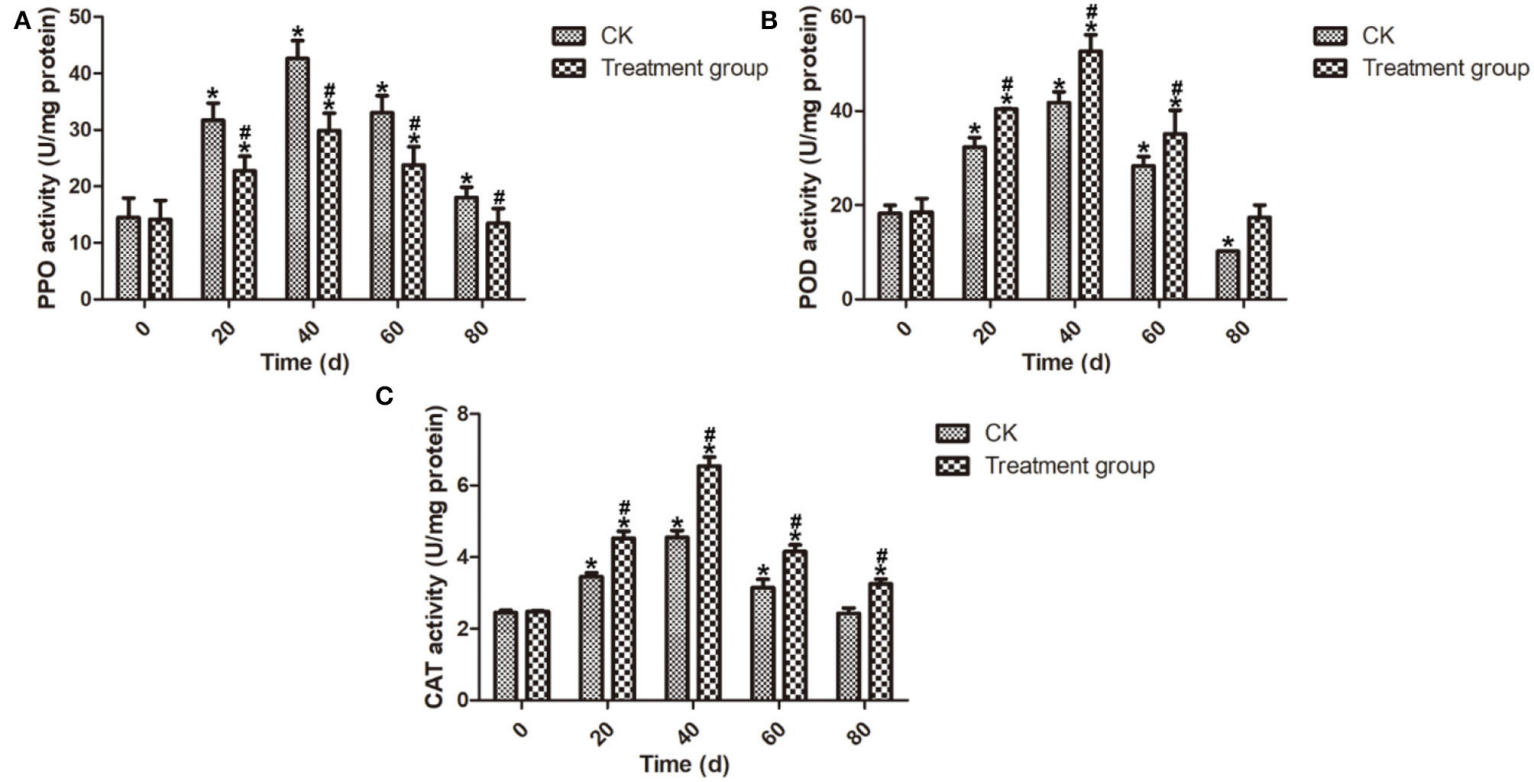
Effects on the enzyme activities of PPO, POD, and CAT of the postharvest blueberries after ozone fumigation treatment. **(A)** Effect on the PPO activity, **(B)** effect on the POD activity, and **(C)** effect on the CAT activity. “*” indicated the decay rates and fruits firmness of ozone fumigation treatment with a significant difference at *p* < 0.05 compared with the decay rates and fruits' firmness at 0 d. “#” indicated the inhibition rates of ozone fumigation treatment with a significant difference at *p* < 0.05 compared with the CK group.

POD was able to increase the accumulation of flavonoid and phenolic compounds to enhance plant disease resistance (Lin et al., 2011). Meanwhile, PPO has been considered a predominant factor leading to the discoloration of fruit (Li et al., 2012). CAT, an important antioxidative enzyme, plays a key role in ROS control (Ng et al., 2005). The mechanism of ozone fumigation treatment to limit decay development in postharvest blueberry fruits was investigated by analyzing the enzyme activity related to defense response. As shown in Figure 5, the PPO, POD, and CAT activities of the postharvest blueberry fruits increased from 0 to 40 d and reached the highest values at 40 d. Meanwhile, it is a remarkable fact that the POD and CAT activities of the postharvest blueberry fruits after ozone fumigation treatment were higher than those of the CK group during the storage. These results suggest that ozone fumigation treatment could enhance the POD and CAT activities to activate the antioxidant defense system in postharvest blueberry fruits. In addition, ozone fumigation could decrease the activity of PPO in postharvest blueberry fruits to inhibit blueberry browning.

## Conclusion

In this study, *Penicillium* sp. was isolated from the rotting postharvest blueberry fruits and identified as genera. Meanwhile, our results indicated that ozone fumigation treatment had an obvious inhibitory effect on *Penicillium* sp. in a dose- and time-dependent manner and thus could prevent the loss of fruit firmness, inhibit the decrease of soluble solids, total phenolics, and anthocyanins assays, and maintain lower PPO activity and higher POD and CAT activities. Our results obtained herein are useful for implementing the ozone fumigation treatment to control the decay of the postharvest blueberry fruits, to extend the storage life of fresh postharvest blueberry.

## Data Availability Statement

The original contributions presented in the study are included in the article/supplementary material, further inquiries can be directed to the corresponding author.

## Author Contributions

WW and JL: methodology, writing—review and editing, and funding acquisition. WW, SC, HC, LR, QL, and SX: data analysis. WW and SC: writing—original draft preparation. All authors have read and agreed to the published version of the manuscript.

## Funding

This research was funded by the Natural Science Foundation of the Department of Education of Guizhou Province [No. qianjiao he KY word (2016)09], the Young Sci-Tech Talents Growth Program from the Department of Education of Guizhou Province [No. qianjiao he KY word (2018)303], the Key Laboratory of Storage of Agricultural Products, Ministry of Agriculture and Rural Affairs, Grant No. KF[2020], and the Research Funding of Guiyang University Support Project, Grant No. GYU-KY-[2022].

## Conflict of Interest

The authors declare that the research was conducted in the absence of any commercial or financial relationships that could be construed as a potential conflict of interest.

## Publisher's Note

All claims expressed in this article are solely those of the authors and do not necessarily represent those of their affiliated organizations, or those of the publisher, the editors and the reviewers. Any product that may be evaluated in this article, or claim that may be made by its manufacturer, is not guaranteed or endorsed by the publisher.
